# Autoimmune Hemolytic Anemia with Autoimmune Hypothyroidism: A Case Report

**DOI:** 10.31729/jnma.8217

**Published:** 2023-07-30

**Authors:** Prakriti Karki, Parikshit Prasai, Vivek Acharya Chetri, Arun Gautam, Robin Maskey

**Affiliations:** 1 B.P. Koirala Institute of Health Science, Ghopa, Dharan, Nepal; 2 Kathmandu Medical College and Teaching Hospital, Sinamangal, Kathmandu, Nepal; 3 Department of Internal Medicine, B.P. Koirala Institute of Health Science, Ghopa, Dharan, Nepal

**Keywords:** *anaemia*, *case reports*, *endocrinology*, *hemolysis*, *hypothyroidism*

## Abstract

Autoimmune hemolytic anaemia is a relatively rare disorder caused by autoantibodies directed against self-red blood cells. Though autoimmune thyroid disease is associated with other autoimmune diseases, only a few cases of Hashimoto's thyroiditis with autoimmune hemolytic anaemia have been reported. We present a case of a 22-year-old woman, a known case of Hashimoto's thyroiditis whose serum demonstrated antibodies against red bloodcells. Bloodinvestigations were done which showed findings suggestive of hemolytic anemia. She was managed with blood transfusion, thyroxine and steroids. Our study may guide physicians toward possible hemolytic anaemia while treating Hashimoto thyroiditis.

## INTRODUCTION

Autoimmune hemolytic anaemia (AIHA) is a rare disorder caused by autoantibodies directed against self-red blood cells (RBC), with an estimated incidence in adults of 0.8-3 per 100,000/year, a prevalence of 17:100,000 and a mortality rate of 11%.^1,2^ Though it has been well established that autoimmune thyroid disease is associated with other autoimmune diseases, only a few cases of Hashimoto's thyroiditis with autoimmune hemolytic anaemia have been reported.^3,4^ We present a case of a 22-year-old lady, with a known case of Hashimoto's thyroiditis complicated by autoimmune hemolytic anemia.

## CASE REPORT

A 22-year-old lady who was a known case of hypothyroidism presented with the chief complaint of generalised weakness of the body for 1 month. Weakness in the body was progressive with increased intensity for 5 days. There was also a history of headaches associated with nausea which was on /off in nature, and a history of yellowish discolouration of the body. There was no significant family history of thyroid disorder. She was under thyroxine for hypothyroidism.

On examination, the patient was well oriented. Lower palpebral conjunctiva was pale, with yellowish pigmentation of the sclera. Her blood pressure was 100/60 mmHg, temperature was 36.4°C, and pulse rate was 90 beats per minute. She had borderline splenomegaly. There were no other signs and symptoms of hypothyroidism.

**Figure 1:**
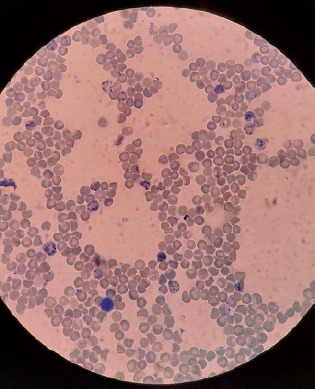
Peripheral blood smear showing reticulocytes.

Blood investigations showed low haemoglobin i.e. 5.3 g/dl, low packed cell volume (PCV) i.e. 17.8%, low RBC count i.e. 1.37 million/ml, and low mean corpuscular haemoglobin concentration (MCHC) i.e. 29.8 g/dl. The erythrocyte sedimentation rate (ESR) was elevated to 51 mm/hr. Likewise, mean corpuscular volume (MCV) and mean corpuscular haemoglobin (MCH) were elevated to 130.1 fl and 38.7 pg respectively. Lactate dehydrogenase (LDH) was elevated to 1118.2 U/L. Her iron profile was normal. A liver function test (LFT) showed total bilirubin of 5.11 mg/dl and conjugated bilirubin of 0.72 mg/dl. The thyroid function test (TFT) was within normal range. Peripheral blood smear showed RBC predominantly with reticulocytes and few macrocytes (Figure 1).

A Direct Coombs test was found to be positive. Moreover, anti-thyroid peroxidase (anti-TPO) antibodies were elevated (>1000 IU/ml) with low C3 and C4 complement levels. Anti-nuclear antibody (ANA) and c-reactive protein (CRP) were within normal. The finding of the ultrasound scan of the abdomen was suggestive of few calculi with a maximum size of stone measuring 5.6 mm with borderline splenomegaly. The clinical scenarios and results from laboratory investigations suggested that the patient was a case of Hashimoto's thyroiditis complicated by autoimmune hemolytic anaemia.

The patient was initially managed with a transfusion of three pints of packed red blood cells. Then, she was started on tablet prednisolone 60 mg per day until her haemoglobin reached more than 10 g/dl, which occurred 15 days after the initiation of steroid therapy. She was clinically and hemodynamically stable at the time of discharge. Then, on discharge, the dose was tapered by 10 mg per week until the dose reached 20 mg per day. On follow-up, her complete blood count (CBC), reticulocyte count and lactate dehydrogenase (LDH) were within the normal range. The dose was then finally tapered by 5 mg per week.

## DISCUSSION

Autoimmune thyroiditis is a common cause of hypothyroidism with a reported incidence of 3.5-5 per thousand per year. Among autoimmune thyroid inflammation, Hashimoto's thyroiditis is the most common type of thyroiditis contributing to 20% of hypothyroidism in non-iodine deficient areas.^5^ The overall incidence of autoimmune hemolytic anaemia from reported studies is 1.77-2.44/100,000 per year.

Among the total AIHA, warm AIHA contributes to almost two-thirds of cases.^6^ Though it has been well established that autoimmune thyroid disease is associated with other autoimmune diseases, only a few cases of Hashimoto's thyroiditis with autoimmune hemolytic anaemia have been reported.^3,4^ In some cases, AIHA has been reported as a component of Evans syndrome.^7^

Hashimoto disease is an autoimmune thyroid disease that is caused by immune-mediated inflammation of the thyroid gland. It is caused by a loss of selftolerance against thyroid antigens. In this condition, a complex interaction between genetic, environmental, and susceptible factors results in autoimmune inflammation and progressive loss of thyroid function. The complex interaction between environmental factors like iodine uptake, smoking, alcoholism, etc, and polymorphism in genetic factors like major histocompatibility gene HLA immune regulatory genes like CTLA 4, thyroid-specific genes, and thyroid peroxisome related genes like TPO, and BACH^2^, results in loss of self-tolerance to thyroid antigens which ultimately lead to autoimmune Hashimoto's thyroiditis.^8-10^ Pathogenesis of autoimmune hemolytic anaemia is complex; no single factor is responsible for the pathogenesis of autoimmune hemolytic anaemia.^11^ It is not well established whether Hashimoto's thyroiditis and autoimmune hemolytic anaemia have common pathogenic mechanisms and have associated temporal associations. In our case, there was a new onset of autoimmune hemolytic anaemia in the background of Hashimoto's thyroiditis. There are limited studies related to the autoimmune hemolytic anemia with autoimmune hypothyroidism.

Hashimoto thyroiditis is diagnosed by the presence of anti-thyroid peroxidase (TPO) antibodies with decreased uptake in thyroid technetium scan.^5^ Positive direct coombs test positive in the background of features of hemolysis like hyperbilirubinemia, and increased reticulocytes help make a diagnosis of autoimmune hemolytic anaemia.^12^ Our case is a typical case of Hashimoto thyroiditis which was diagnosed 2 years back by the presence of anti-thyroid peroxidase (TPO) antibodies with decreased uptake in thyroid technetium scan. While on treatment she presented to our centre with clinical and laboratory findings consistent with autoimmune hemolytic anaemia without any other autoimmune disease and triggering factors. This combination may guide us toward possible hemolytic anaemia while treating Hashimoto thyroiditis. Management of autoimmune hemolytic anemia is well established and can be done by steroids and refractory cases are managed by rituximab.^12^ In our case, the patient responded well to steroids with a combination of thyroxine.
